# The relationship between the bone characters obtained by CBCT and primary stability of the implants

**DOI:** 10.1186/s40729-014-0003-x

**Published:** 2015-02-12

**Authors:** Masahiro Wada, Yasutane Tsuiki, Tohru Suganami, Kazunori Ikebe, Motofumi Sogo, Ikuhisa Okuno, Yoshinobu Maeda

**Affiliations:** Department of Prosthodontics, Gerodontology and Oral Rehabilitation, Osaka University Graduate School of Dentistry, 1-8 Yamadaoka, Suita, Osaka 565-0871 Japan

**Keywords:** Primary stability, CBCT, Voxel value

## Abstract

**Background:**

The aim of this study is to investigate the correlation between the thickness of the cortical bone or the voxel values that are obtained by cone beam CT (CBCT) and the insertion torque values (ITVs) or the implant stability quotient (ISQ) values.

**Methods:**

A pig's ilium was used as the implant placement site. The implants used in this study were two kinds of diameters (3.8 mm, 5.0 mm) and two kinds of lengths (7.0 mm, 12.0 mm) having a general threadlike shape with a mechanically polished surface. To measure the bone density and the cortical thickness around the implants accurately, the CBCT scanning was performed immediately just after the formation of the implant cavity. The initial stabilities were evaluated by the ITVs and the ISQ values. The bone density and cortical thickness around the implants were measured by an implant simulation software (Landmarker ver. 5.0 with special specifications for this study). The relationships of the thickness of the cortical bone and the voxel values with the ITVs and the ISQ values were analyzed using Pearson's correlation coefficient. To evaluate the influence on the ITVs and the ISQ values among multiple factors, multiple regression analysis was performed. *P* < 0.05 was considered statistically significant.

**Results:**

A significant positive correlation was found between the thickness of the cortical bone and the ITVs or the ISQ values in all kinds of implants. In addition, a significant positive correlation was also found between the voxel values and the ITVs. From the multiple regression analysis, the thickness of the cortical bone and the voxel values had a positive influence on the ITVs and the ISQ values. In addition, the length of the implant had a positive influence on the ISQ values at the 3.8-mm-diameter implant.

**Conclusions:**

In this limited study, there were correlations between the thickness of the cortical bone or the voxel values obtained from the CBCT scanning and the implant stabilities. Besides, it was confirmed that the thickness of the cortical bone, the voxel value, and the implant length had positive correlations with the ITVs and the ISQ values.

## Background

The primary stability of an implant at the time of placement is considered as one of the key factors for clinical success of implant treatment [1-6]. Orenstein et al. reported that implants that were appropriately stabilized without any mobility at the time of placement had a significantly high survival rate compared with those that were not [7].

The evaluation of the primary implant stability is usually performed after placement. Some of the main methods include mobility test, resonance frequency analysis, and the measurements of the removal torque values and the insertion torque values (ITVs). In particular, the measurement of the removal torque values is an objective evaluation method, but its clinical application is difficult because it is an irreversible and invasive method. Mobility test is useful for the evaluation of an implant whose osseointegration was surely obtained, but there is a possibility that the primary stability could decrease by the impact of the tapping head. On the other hand, the measurement of ITVs and the measurement of implant stability quotient (ISQ) values by using a resonance frequency analyzer are non-invasive, convenient, and objective evaluation methods. Therefore, these methods are used for evaluation in various researches investigating the primary stability including immediate loading implants [8-11].

The primary stability is significantly affected by bone quality. Herrmann conducted a study of the prognosis for as long as more than 5 years and reported that poor bone quality and quantity had a major impact on the long-term failure rate of implants [12]. Jaffin observed the prognosis of implants for 5 years after providing the final restoration [13]. As a result, the failure rate of the implants was 3% when the implant was placed in the alveolar bone having a thick cortical bone or otherwise a dense spongy bone even if the cortical bone was thin, whereas it was 35% when the implant was placed in the alveolar bone having both a thin cortical bone and a sparse spongy bone.

Some of the methods to evaluate the bone quality that influences the primary implant stability have already been applied in a clinical practice. Lekholm and Zarb classified bone density into four types in terms of radiography, with the thickness of the cortical bone and the density of the spongy bone as the indexes [14]. This classification method is accepted most commonly at present but is problematic with accuracy and reproducibility because it is a subjective evaluation. On the other hand, Misch classified CT values (Hounsfield unit) into five steps (D1: >1,250 HU; D2: 850 to 1,250 HU; D3: 350 to 850 HU; D4: 150 to 350 HU; D5: <150 HU) to evaluate the bone quality [15]. The CT value is the value obtained by multi-detector CT (MDCT) and is defined as the relative value of the X-ray attenuation coefficient of the object for water, with the X-ray attenuation by water defined as zero. Today, this classification has been used for the evaluation of the bone quality because it is an objective method compared with that of Lekholm and Zarb.

Turkyilmaz et al. placed 24 implants in human dry bones and calculated CT values using MDCT images before surgery to examine correlations with the ITVs and the ISQ values [16]. They reported that there were significant correlations of CT values with both ITVs and ISQ values and that bone density (CT value) was one of the factors that had an influence on the primary implant stability.

In recent years, cone beam CT (CBCT) has been used for preoperative diagnosis in implant treatment. CBCT is superior for its high definition, reduction of the exposure dose, low cost, and usability compared with MDCT [17-21]. However, CBCT does not have a linear relationship compared with the CT values obtained by MDCT, and therefore, it is considered difficult to evaluate bone density quantitatively [22-24]. The major causes arise from the lack of calibration of X-rays, the localized imaging area that allows various external anatomical structures, and too many scattered radiations.

It is therefore considered that MDCT is appropriate for the precise evaluation of bone density. However, since the use of CBCT is spreading rapidly among general practitioners, it is clinically of great significance to predict the primary stability after implant placement using the information obtained by CBCT. In addition, in late years, CBCT or the calibration software which can convert the voxel values into CT values is developed.

We therefore investigated in this study the correlation between the thickness of the cortical bone or the voxel values that are obtained by CBCT and the ITVs or the ISQ values.

## Methods

### Bone models

**Figure 1:**
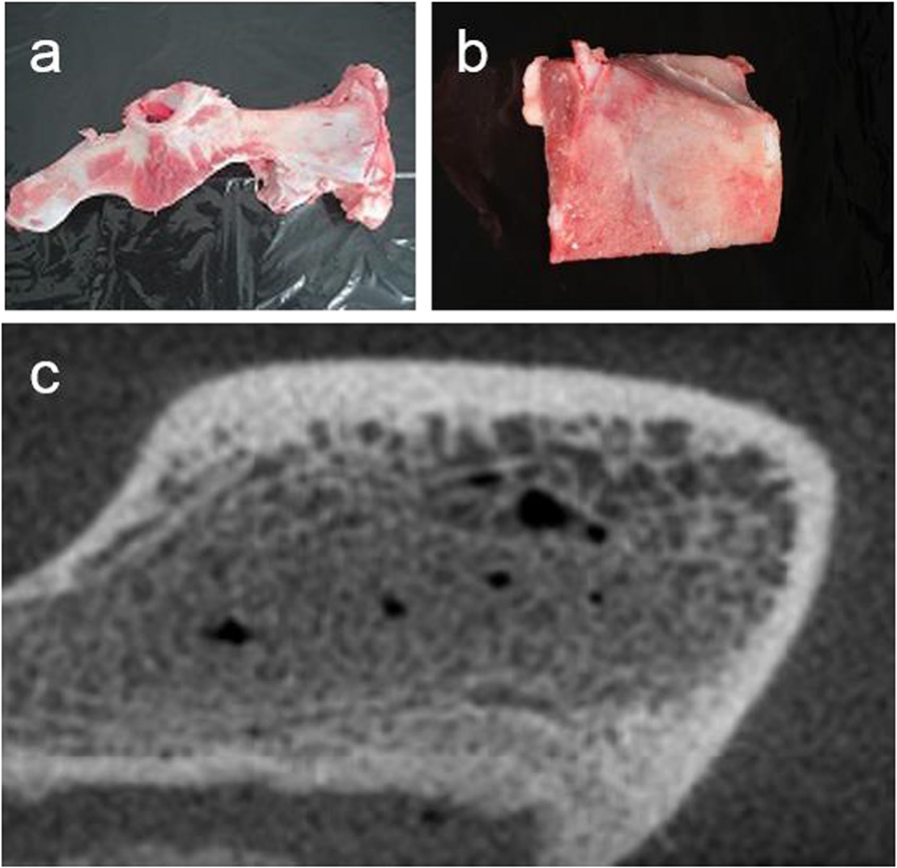
**The bone model in this study (a pig's ilium). (a)** The whole picture of the ilium. **(b)** The flat part of the posterior margin of the ilium. **(c)** The CT image of the ilium.

A flat part of a pig's ilium was used as the implant placement site to secure as vertical implant placement and an equal depth of insertion to the bone surface as possible (Figure 1).

### CBCT scan

GXCB-500® (GENDEX, Des Plaines, IL, USA) was used as the CBCT device to obtain almost identical voxel values to the CT values that could be obtained from MDCT. Scanning conditions were as follows: the tube current, 5 mA; the tube voltage, 120 kV; the field of view (FOV), 85-mm diameter to secure a complete size for scanning; and the voxel size, 0.1 mm^3^. In addition, the scanning was performed by immersing the samples in a polypropylene container with water in it to create as similar scanning environment as possible to the one that had soft tissues around the bone model. Of note, the scanning was performed immediately just after the formation of the implant cavity to accurately measure the voxel values around the inserted implants.

### Implants

**Figure 2:**
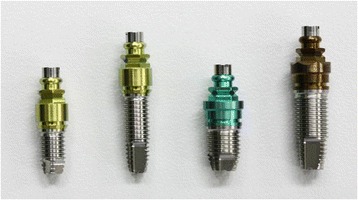
**The implants in this study.** Two kinds of diameters (3.8 mm, 5.0 mm) and two kinds of lengths (7.0 mm, 12.0 mm) having a general threadlike shape with a mechanically polished surface.

External Hex Implants for laboratory use (SETiO®, GC Company, Tokyo, Japan) having a general threadlike shape with a mechanically polished surface were used. They were two kinds of diameters (3.8 mm, 5.0 mm) and two kinds of lengths (7.0 mm, 12.0 mm) consisting a total of four groups, and 25 pieces of implants were used in each group (Figure 2).

### Formation of the implant cavities

**Figure 3:**
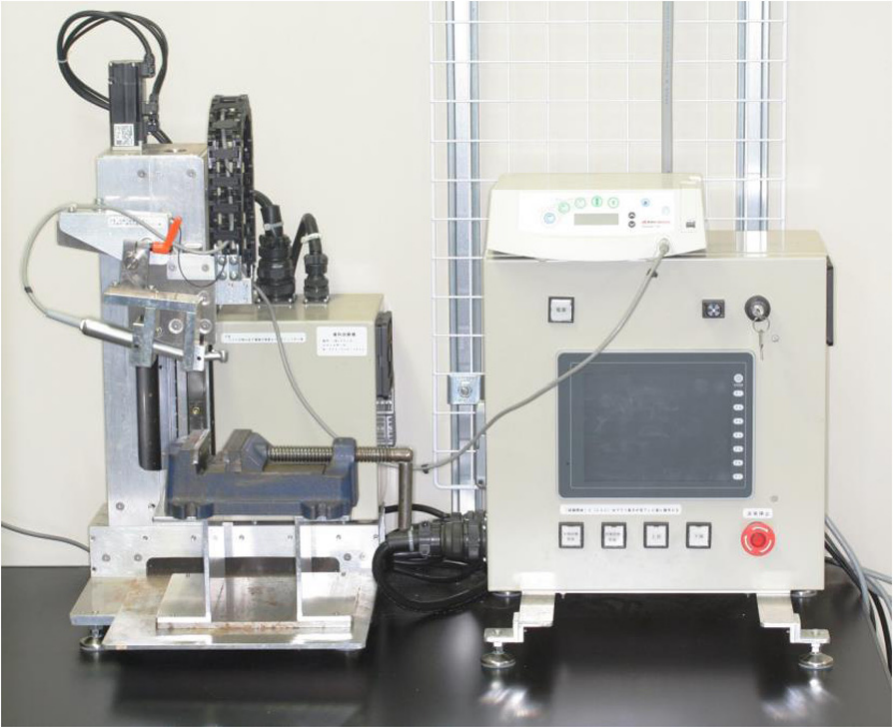
**The special implant cavity-forming device.** This device is able to adjust the up-and-down movement speed and the rotation speed of the drill.

When forming an implant cavity, it is necessary to move the drill back and forth along the insertion direction. This process is usually done by an operator with freehand, but it is likely to cause unevenness in the diameter of the implant cavity. Therefore, an implant cavity-forming device was used to prevent this in our experiment (Figure 3). This device is able to adjust the up-and-down movement speed and the rotation speed of the drill. In this study, the implant cavity was constructed at a moving speed of 5 mm/s that was close to the actual up-and-down movement speed obtained from the preliminary experiment and at 800 rpm according to the manufacturer's protocol. It was also confirmed in the preliminary experiment that using this device would significantly reduce the unevenness of the diameter of the implant cavity.

All the procedures of forming an implant cavity were unified as follows according to protocols. Firstly, the implant cavity was constructed by using a 2.0-mm-depth drill after marking the implant site using a guide drill. Secondly, a 3.1-mm pilot drill and a 3.1-mm twist drill were used to form a cavity for an implant with 3.8-mm diameter, and a 4.3-mm pilot drill and a 4.3-mm twist drill were used to form a cavity for an implant with 5.0-mm diameter. Of note, this experiment was performed under a non-irrigation environment because the rise of temperature would not be problematic.

### Placement of an implant and the measurement of the implant stabilities

Handy Type Torque Meter (HTG2-200NC, IMADA-SS Corp, Aichi, Japan) was used for the placement of an implant, and the maximum torque values were measured. The specifications of the Handy Type Torque Meter were as follows: the measurement unit, Ncm; the accuracy, within ±0.5% FS; the measurement maximum torque, 200 Ncm; and the minimum resolution function, 0.1 Ncm. Thus, the device had enough coverage of the torque measurement in this study (96.0 Ncm at maximum).

Osstell Mentor® (Integration Diagnostics AB, Gôteborg, Sweden) was used as a resonance frequency analysis device to measure the ISQ values. The accompanying smart peg (type 1) was attached to the implant for the measurement at every 90°, and the average value was calculated.

### Measurement of the voxel values and the thickness of the cortical bone around implants

**Figure 4:**
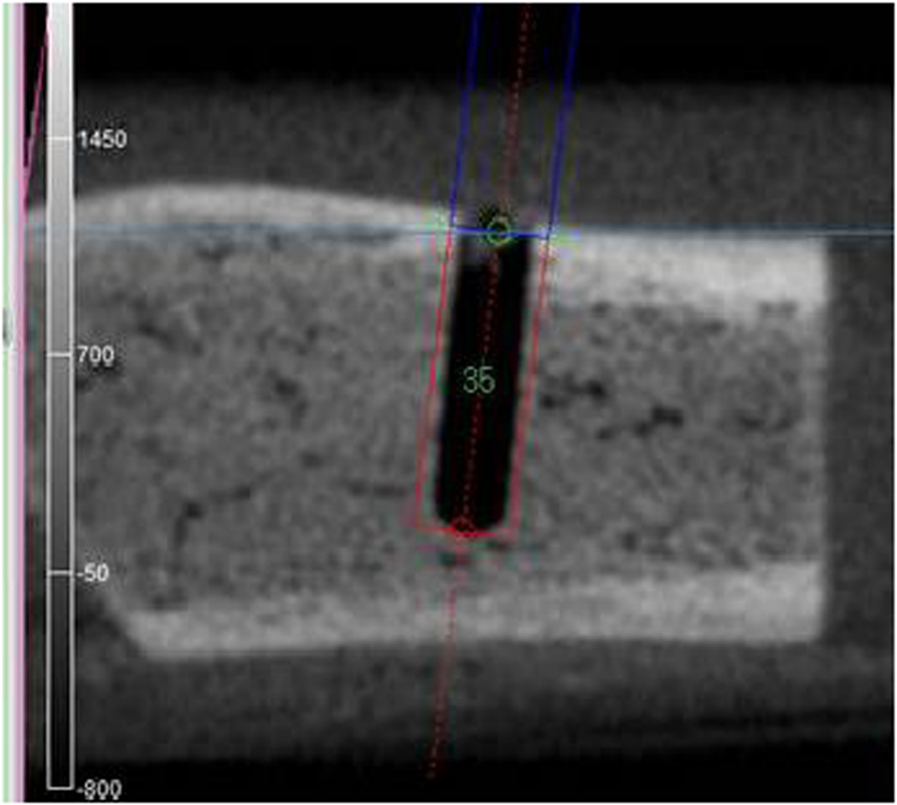
**Setting the evaluation site.** An implant placement simulation software (Osaka Landmarker ver. 5.0 with special specifications for study purposes, iCAT, Osaka, Japan) was used as the image analysis software. The virtual implant was placed in the implant cavity by simulation.

**Figure 5:**
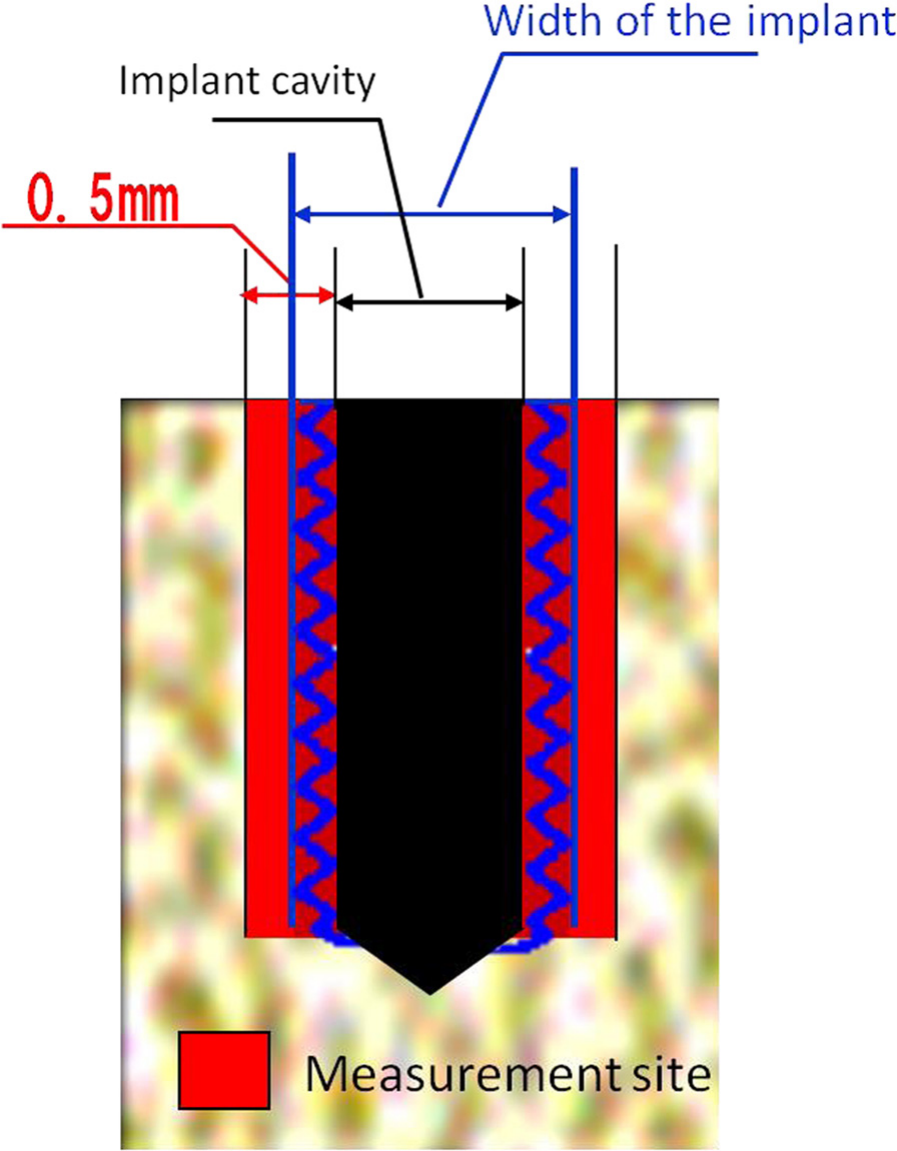
**Measurement site of the voxel values.** The width of the measurement site was defined as 0.50 mm, i.e., from 0.25 mm inside (the to-be-compressed area at the time of placement) to 0.25 mm outside (the same width of the aforementioned) of the virtual implant.

An implant simulation software (Landmarker ver. 5.0 with special specifications for study purposes, iCAT, Osaka, Japan) was used for analysis. The evaluation site was selected on the smallest area that was as adjacent to the implant as possible so as not to include the area that was immune to the primary stability. Specifically, the width of the measurement site was defined as 0.50 mm, i.e., from 0.25 mm inside (the to-be-compressed area at the time of placement) to 0.25 mm outside of the virtual implant. The area adjacent to the bottom of the implant was excluded from the measurement site because the maximum torque value and the ISQ value were both subject to the lateral force a great deal (Figures 4 and 5). Then, the voxel values of the measurement site were extracted into the comma-separated values (CSV) files. Subsequently, the average of the voxel values of the measurement site was calculated and defined as the voxel value of the whole bone around the implant. Additionally, the average of the voxel values was calculated at every 0.1-mm depth from the surface of the bone to determine the thickness of the cortical bone from the voxel values. The threshold of the voxel value for the border of the cortical bone and the spongy bone was decided to be 350, which had a strong correlation (Pearson's correlation coefficient, 0.897) with the actual measurement in the preliminary examination.

### Statistical analysis

The relationships of the thickness of the cortical bone and the voxel values with the ITVs and the ISQ values were analyzed using Pearson's correlation coefficient. Then, multiple regression analysis was performed using the ITVs or the ISQ values as the dependent variable and using the thickness of the cortical bone, the voxel value, and the length of the implant as the independent variables to evaluate the influence on the ITVs and the ISQ values among multiple factors. The statistical analyses were performed using the SPSS ver. 22 software (SPSS Co., Chicago, IL, USA). *P* < 0.05 was considered statistically significant.

## Results

### Relationship between the thickness of the cortical bone and voxel values with implant stabilities

A significant positive correlation was found between the thickness of the cortical bone and ITVs or ISQ values in all kinds of implants. In addition, a significant positive correlation was also found between the voxel values and ITVs. On the other hand, in the relationship between the voxel values and ISQ values, we cannot confirm a correlation of the implant of 5.0 mm in width and 12.0 mm in length (Table 1).

**Table 1:** **Correlation between bone factors and stability factors**

**Bone factors/stability factors**	**Diameter (mm)**	**Length (mm)**	***r***	***P***	***n***
Thickness of the cortical bone/ITVs	3.8	7	0.744**	<0.001	24
		12	0.560**	0.005	23
	5	7	0.815**	<0.001	25
		12	0.760**	<0.001	24
Thickness of the cortical bone/ISQ	3.8	7	0.831**	<0.001	24
		12	0.409	0.052	23
	5	7	0.707**	<0.001	25
		12	0.426*	0.038	24
Voxel values/ITVs	3.8	7	0.601**	0.002	24
		12	0.745**	<0.001	23
	5	7	0.850**	<0.001	25
		12	0.667**	<0.001	24
Voxel values/ISQ	3.8	7	0.684**	<0.001	24
		12	0.447*	0.032	23
	5	7	0.695**	<0.001	25
		12	0.270	0.202	24

ITVs, insertion torque values. **P < 0.01; *P < 0.05.

### Influence of the implant of 3.8 mm in width on the ITVs and the ISQ values of each factor

Multiple regression analysis was performed using the ITVs or the ISQ values as the dependent variable and using the thickness of the cortical bone, the voxel value, and the length of the implant as the independent variables. The standardized partial regression coefficients of the thickness of the cortical bone and the voxel value were 0.400 and 0.459, respectively, which turned out to be significant explanatory variables. However, that of the length of the implant did not become an explanatory variable. When using the ISQ values as the independent variable, the standardized partial regression coefficients of the thickness of the cortical bone, the voxel value, and the length of the implant were 0.326, 0.304, and 0.420, respectively, all of which became significant explanatory variables. From these results, it was confirmed that the thickness of the cortical bone and the voxel values had a positive influence on ITVs, while the thickness of the cortical bone, the voxel value, and the length of the implant had a positive influence on the ISQ values (Table 2).

**Table 2:** **Statistical analysis of the results of the multiple regression analysis of the 3.8-mm-width implant**

Dependent variable = ITVs	(*n* = 47)
Independent variables	Standardized partial regression coefficient (*P* value)
Thickness of the cortical bone	0.400 (=0.02)
Voxel value	0.459 (<0.01)
Length of the implant	0.005 (=0.97)
	*R* ^2^ = 0.633
Dependent variable = ISQ values	(*n* = 47)
Independent variables	Standardized partial regression coefficient (*P* value)
Thickness of the cortical bone	0.326 (=0.04)
Voxel value	0.304 (<0.01)
Length of the implant	0.420 (<0.01)
	*R* ^2^ = 0.593

### Influence of each factor of the implant of 5.0 mm in width on ITVs and the ISQ values

The standardized partial regression coefficients of ITVs for the thickness of the cortical bone and the length of the implant were 0.408 and 0.526, respectively, which became significant explanatory variables. In addition, those of the ISQ values for the thickness of the cortical bone and the length of the implant were 0.440 and 0.750, respectively, which also became significant explanatory variables. From these results, it was confirmed that the thickness of the cortical bone and the length of the implant had a positive influence on ITVs and the ISQ values. However, the voxel value of ITVs and that of the ISQ values failed to become explanatory variables (Table 3).

**Table 3:** **Statistical analysis of the results of the multiple regression analysis of the 5.0-mm-width implant**

Dependent variable = ITVs	(*n* = 49)
Independent variables	Standardized partial regression coefficient (*P* value)
Thickness of the cortical bone	0.408 (=0.04)
Voxel value	0.365 (=0.10)
Length of the implant	0.526 (<0.01)
	*R* ^2^ = 0.638
Dependent variable = ISQ values	(*n* = 49)
Independent variables	Standardized partial regression coefficient (*P* value)
Thickness of the cortical bone	0.440 (=0.02)
Voxel value	−0.060 (=0.98)
Length of the implant	0.750 (<0.01)
	*R* ^2^ = 0.836

## Discussion

### Measurement of the cortical bone thickness and the voxel values

Ikumi used a MDCT scan for actual patients to calculate the CT values of the 1-mm surrounding area of the planned implant placement site using implant simulation software [25]. However, it is likely that the precise measurement cannot be performed in the case where the actual implant cavity was formed off the planned implant site because the density of the bone around the planned implant site was evaluated by preoperative simulation.

Therefore, we evaluated the bone quality 0.5 mm surrounding the implant, which was thought to have a strong effect on the primary stabilities, by CBCT scanning after forming the implant cavities.

Nkenke used an axial image to define the thickness of the cortical bone from the average thickness of the cortical bone around the implant measured by eye estimation and then evaluated its relationship with ITVs [26]. Whereas in this study, we measured the thickness of the cortical bone by setting the threshold for the voxel values around the implant for the purpose of securing reproducibility of the measurement of the thickness of the cortical bone. The threshold that we set was determined in the preliminary experiment so that it would highly correlate with the measurement values of the thickness of the cortical bone measured by eye estimation. Conversely, the limitation of this study is that the CBCT device is lacking some precision for estimation of bone density compared to quantitative computed tomography.

### Relationship between the thickness of the cortical bone and the voxel values measured by CBCT and the primary stability of the implant

To date, there are several studies that investigated the relationship between the thickness of the cortical bone and the primary stability of the implant. Motoyoshi placed a total of 87 mini-implants, which were used as anchors for orthodontic treatment, in the buccal alveolar bone in the molar region of actual patients [27]. As a result, they reported that they found a positive significant correlation between the thickness of the cortical bone measured by using medical CT images and the torque value at the time of placement. Roze measured the thickness of the cortical bone of the jawbones in three human bodies using μCT and placed a total of 22 implants [28]. According to this report, there was a significant correlation between the thickness of the cortical bone and the ISQ values measured immediately after the implant placement. Furthermore, Isoda reported a significant correlation between the bone quality around the implants and the implant stabilities.

Although this study is different from the previous studies in that the thickness of the cortical bone and bone density were determined from the voxel values calculated by CBCT, a significantly positive correlation of the thickness of the cortical bone and the voxel values with ITVs and ISQ values was confirmed as in the previous studies. Furthermore, the multiple regression analysis with the ITVs and the ISQ values as the dependent variables showed that the thickness of the cortical bone as well as the voxel values had a positive influence.

Based on these results, it was revealed that the thickness of the cortical bone obtained from CBCT had a correlation with the ITVs and the ISQ values which are the indexes of the primary stability of the implant. It was also revealed that the voxel values correlated with ITVs and ISQ values. It is desirable to be able to infer the primary stability at the time of implant placement not only from the thickness of the cortical bone but also from the voxel values, if the cortical bone cannot be observed on the CT images of the extraction socket immediately after or at a certain period of time after the tooth extraction (until the maturation of the bone). Conversely, there was no statistical correlation between the voxel values and ISQ values among 5-mm-diameter, 12-mm-length implants. These ISQ values were high enough regardless of bone densities, and this caused that the correlation did not confirm between voxel values and ISQ values.

In addition, these results were confirmed by using mechanically polished surface implants. Therefore, it is thought that primary stability becomes more stable by using rough-surface implants.

## Conclusions

In this limited study, there was a correlation between the thickness of the cortical bone or the voxel values obtained from the CBCT scanning images prior to the implant placement and the implant stabilities. Besides, it was confirmed that the thickness of the cortical bone, the voxel value, and the length of the implant had positive correlations with the ITVs and that the thickness and length had positive correlations with the ISQ values.
